# New Appliances and Things Medical

**Published:** 1896-04-11

**Authors:** 


### NEW APPLIANCES AND THINGS MEDICAL.

[We shall be glad to receive, at our Office, 428, Strand, London, W.C., from the manufacturers, specimens of all new preparations and appliances, which may be brought out from time to time.]

### THE BALL NOZZLE SYRINGE.

(British-American Ball Nozzle Company, 52, Oxford Street, W.)

The principle of the ball nozzle is one of the most interesting in hydrodynamics, and one of the most difficult of explanation. The discovery, which was an accidental one—indeed, it could scarcely have been predicted from scientific reasoning—is as follows: If a fluid stream is allowed to escape through a cup-shaped orifice, and a spherical ball be placed in the cup so as to fill it more or less completely, the ball, instead of being projected to a distance by the force of the stream, remains *in situ*, and causes a spread of the fluid in a sort of thin film between the surface of the cup and that of the ball. The stream as it leaves the cup continues for some distance in the same form, *i.e.*, that of a cylindrical film, and at a distance which varies with the force of the stream and the size of the nozzle, breaks up into the finest of sprays. The applications of such a principle are obviously unlimited, but up to the present the chief commercial uses to which it has been put has been in fire brigades and for the purposes of horticulture. So valuable a discovery obviously had its uses in surgery, and the profession have not been slow to apply it to douches and sprays of all kinds—an ordinary enema provided with a ball nozzle is clearly an ideal instrument for the washing out of canals and passages. The stream is gentle and equable, and an immense improvement on that of the older forms. Messrs. Maw, Son, and Thompson are the wholesale agents in England, and the price for a bulb enema in grey rubber is 7s. 6d., in seamless red rubber 9s. 6d.

### EXTRACTUM SANTALI SOLUBILE AND LIQUOR PICIS AROMATICUS.

(John Bell and Co., 225, Oxford Street, W.)

Extractum Santali Solubile is a soluble liquid extract of white sandal wood, and it has been prepared by John Bell and Co. as a substitute for the ordinary sandal wood oil, which has a high reputation in the treatment of chronic inflammation of the mucous membranes. A continued use of the oil is so often followed by gastric disturbance that it is seldom found possible to persevere with its administration. The extract of the wood, as prepared by the manufacturers, is a dark coloured liquid, which can be diluted with water without precipitation. It possesses the astringent properties of other sandal wood preparations. It is neutral in reaction, and is stated to contain a solution of all those active principles to which sandal wood oil owes its reputation. As far as taste is concerned it has great advantages over the oil.

Liquor Picis Aromaticus.

Solutions of tar, made by the destructive distillation of various pine woods contain such bodies as guaiacol, creasol, and pyrocatechin, substances which have valuable properties in the treatment, chronic pulmonary catarrhs and phthisis. Ordinary tar water contains but small quantity of these substances in solution, and hence the necessity for large and unpleasant doses. This new preparation of John Bell and Co. need be taken in doses of one drachm only, and the taste being skilfully concealed by pleasant aromatic bodies, it constitutes a valuable substitute for the tar preparation hitherto in use for pulmonary complaints.

### SOLUBLE FOOD, COD LIVER OIL, &c.

(Carnrick and Co. (Limited), 24, Hart Street, Bloomsbury, London.)

Carnrick's soluble food, when prepared with the required quantity of water, constitutes an adequate and sufficient diet for infants, closely simulating in its composition human milk. By reason of its constant composition and the ease with which it can be prepared and digested, it is a food which should have much to recommend it to those responsible for the rearing of children.

Cod Liver Oil Milk.

This is a really valuable preparation of cod liver oil which, as we can vouch for from practical experience, can be readily taken by those who are absolutely unable to take the oil in its undisguised form. Another preparation which has been forwarded us by the same firm, and which is prepared by the Maltine Manufacturing Company (Limited) is maltine with coca wine. Each fluid ounce of this liquid is stated to contain 30 grs. of Erythroxylon coca; the combination of a fine malt extract with so powerful a tonic renders this new preparation one of the most useful in the treatment of the debility and mal-nutrition.

